# Trends in Unprocessed Red and Processed Meat Consumption in the Mexican Population, 2006–2020

**DOI:** 10.1016/j.cdnut.2025.107598

**Published:** 2025-11-12

**Authors:** Kaela Connors, Lindsay M Jaacks, Peter Alexander, Juan A Rivera, Carolina Batis

**Affiliations:** 1 School of Geosciences, University of Edinburgh, Edinburgh, United Kingdom; 2 Global Academy of Agriculture and Food Systems, University of Edinburgh, Edinburgh, United Kingdom; 3 Center for Health Systems Research, National Institute of Public Health, Cuernavaca, Mexico; 4 Center for Nutrition and Health Research, National Institute of Public Health, Cuernavaca, Mexico

**Keywords:** unprocessed red meat, processed meat, consumption trends, sustainable and healthy diets, Mexico

## Abstract

**Background:**

Meat consumption trends in Mexico are poorly understood, and this may have implications for Mexico’s diet-related disease burden and the sustainability of diets.

**Objectives:**

We assessed trends in protein-rich food groups and the contribution of unprocessed red and processed meat to nutrient intake.

**Methods:**

We used nationally representative (all ages) dietary information from the Mexican National Health and Nutrition Survey, collected using a semiquantitative food frequency questionnaire in 2006, 2012, 2016, 2018, and 2020. We tested for time trends and sociodemographic differences in daily consumption using survey-weighted generalized linear regression, adjusting for total energy intake. The contribution to nutrient intake was assessed from a 2016 24-h dietary recall.

**Results:**

Energy-adjusted unprocessed red meat consumption decreased slightly from 2006 to 2020, but processed meat remained unchanged [change in daily consumption (g) per survey cycle (95% confidence interval) *β* = −0.28 (−0.49, −0.08) and 0.01 (−0.06, 0.09), respectively]. Among 6–19 y olds, unprocessed red and processed meat consumption marginally increased. Other protein-rich foods such as seafood, legumes, nuts, and seeds decreased over time, whereas poultry and dairy increased. Egg consumption remained constant. Unprocessed red and processed meat contributed substantially to per capita heme iron intake (23% and 26%, respectively) and vitamin B12 intake (17% and 9.8%, respectively).

**Conclusions:**

Processed meat consumption is well-above dietary targets and has not declined in Mexico, with modest increases among young people, posing potentially negative health and environmental consequences. Meat was a major contributor to heme iron and vitamin B12, but less so (<10%) for other essential nutrients. Therefore, policies to encourage meat reduction, particularly processed meat, are unlikely to adversely affect nutritional status.

## Introduction

High consumption of unprocessed red and processed meat has been linked to negative consequences for both human health and the environment [[1], [2], [3], [4], [5], [6]]. Recognizing this, the 2023 Mexican Dietary Guidelines recommend reducing unprocessed red meat consumption and avoiding processed meat altogether to achieve health and sustainability goals [7]. The guidelines encourage high consumption of fruits, vegetables, beans, and whole grains, which is a major shift from the current diet characterized by high intake of animal-sourced and ultraprocessed foods [[8], [9], [10]].

Unprocessed red and processed meat consumption exceeds healthy and sustainable dietary targets in the Mexican population [11,12]. Specifically, less than half the Mexican adult population is within the recommended consumption limit for unprocessed red meat, and processed meat consumption is well-above (4 daily servings compared with 0 recommended) [10]. However, this is based on cross-sectional dietary information from 2016 [11,12] and 2020–2023 [10], with limited knowledge of these trends over time. There are 2 previous studies that have analyzed consumption trends of these foods over time in Mexico [13,14]. The first did not disaggregate unprocessed red and processed meat from animal-source foods but did find a significant increase in animal-sourced foods comparing average consumption in 2012–2016 [13]. The second study found a significant increase in energy contribution from processed meat in children but not in adolescent girls nor females of reproductive age, and found no changes in red meat comparing 1999–2012 [14]. However, trends across multiple time points and in the general Mexican population are still undetermined.

Here, we aimed to quantify trends in consumption of unprocessed red and processed meat and other protein-rich food groups from 2006 to 2020 in a nationally representative sample of the Mexican population. Given segments of the population—particularly lower income and indigenous groups—may be at risk of insufficient nutrient intake [15], we also sought to better understand the contribution of meat to nutrient intake and the types of meat consumed.

## Methods

### Study population and sample size

We used nationally representative dietary information from the Mexican National Health and Nutrition Survey (ENSANUT, by its Spanish acronym) [16]. ENSANUT uses a multistage probability design to sample the civilian, noninstitutionalized population of Mexico. Informed consent was obtained from individuals (≥18 y), and consent for younger participants (<18 y) was obtained from the parent or caretaker. Multiple individuals per household were interviewed based on age group. Information was obtained through face-to-face interviews by trained health personnel, and interviews occurred between July and November, with small differences between the years. This means the diet would be temporally comparable across individuals, while reflecting some seasonal variation in foods, although it may not accurately reflect the usual intake of foods eaten throughout the entire year. An exception for this was the 2016 cycle, which took place between May and September. Description of the sampling frame and methods has been described elsewhere [16].

We used 6 rounds of surveys: 2006 (*n =* 38,775), 2012 (*n =* 6729), 2016 (*n =* 14,537), 2018 (*n =* 23,516), and 2020 (*n =* 2033), and included all ages (1−111 y) (Supplemental Table 1). The sample size for 2020 is smaller than other cycles, given that 2020 was the first of 5 surveys from 2020 to 2025, ENSANUT–Continua, that were administered annually for up-to-date, rapid nationally representative epidemiologic surveillance on health, nutrition, and health service provision, especially in light of the COVID-19 pandemic [17]. Therefore, more frequent, annual surveys resulted in reduced sample sizes for surveys in ENSANUT–Continua. The data were cleaned for implausible intake of food quantity, energy, and nutrients according to methods applied to ENSANUT previously published elsewhere [18]. We additionally excluded participants who reported consuming ≤400 kcal/d (*n =* 668) and ≥4000 kcal/d (*n =* 1736) or those who were either pregnant or lactating (*n =* 1083), given the distinct nutritional needs in this population that may not reflect long-term, standard dietary behaviors [19]. Additionally, the number of pregnant and lactating females in ENSANUT is limited, and the sampling frame is not designed to represent this small subpopulation.

We additionally utilized a 24-h dietary recall, including all ages (1–97 y) in 2016, with a sample size of *n* = 4070.

All surveys are representative at the national, urban, rural, and federal entity level with similar designs, which enable their comparability over time [17].

### Dietary information

Dietary data were collected using a semiquantitative food frequency questionnaire (FFQ) with a 7-d recall period, which included 101 food items in 2006, and from 2012 onward it was expanded to 140 food items [20]. We ensured comparability of items across FFQ versions. Briefly, some examples of differences in the food groups evaluated: unprocessed red meat, processed meat, eggs, poultry, and fish. The items were directly comparable in the 101 compared with the 140-item FFQ. The main variations were found in dairy, in which items were comparable but expanded on. For example, in 2006, there was 1 food-item called “cheese,” whereas in 2012–2020 this was expanded to different types of cheeses consumed in Mexico, such as “panela cheese” or “hard cheese (i.e., chihuahua, manchego).” However, because FFQs capture habitual consumption, estimations across both are comparable [19]. The FFQ has previously been validated for estimating energy and nutrient intakes in Mexican adolescents and adults against two 24-h dietary recalls as the reference method, finding that the FFQ tended to estimate slightly higher energy and nutrient values [20]. Pearson correlation coefficients for absolute nutrient intakes between both instruments indicated good validity (∼ 0.36) in comparison with other validation studies in both age groups.

We additionally utilized information from a 24-h dietary recall to assess the contribution of unprocessed red and processed meat to nutrient intake, granted that the recall captures more granular information on food items, and thereby nutrient intake. Moreover, 24-h dietary recalls are better at approximating population mean consumption [19], which was our objective in estimating nutrient contribution. Whereby FFQs are better at estimating usual consumption, therefore, more appropriate for the study of trends [19].

Food items from the FFQ were categorized into the following 7 protein-rich food groups: *1*) unprocessed red meat, *2*) processed meat, *3*) poultry and offal (herein, poultry), *4*) seafood, *5*) dairy, *6*) eggs, and *7*) legumes, nuts, and seeds (here, legumes). Unprocessed red meat refers to “unprocessed mammalian muscle meat” such as beef, veal, pork, and lamb [21]. Whereas, processed meat refers to meat (which in this case includes both red and white meat) that has undergone processing such as “salting, curing, fermentation, smoking” for the purposes of enhancing flavor or preservation [21]. We disaggregated mixed dishes into their individual ingredients using a Mexican National Institute of Public Health (INSP, by its Spanish acronym) recipe file (unpublished data). The categorization of other protein-rich foods was based on those suggested as potential substitutes to unprocessed red and processed meat in the Mexican Dietary Guidelines 2023 [7]. We converted consumption to daily consumption (grams) based on specified portion sizes and reported servings per week. Total meat was combined with daily unprocessed red and processed meat consumption. We calculated daily total energy intake according to the sum of the daily amount consumed of each food by the participant across all survey cycles.

For the 2016 24-h recall, we classified unprocessed red and processed meat according to the same criteria as the FFQ. Nutrients and energy intake were obtained from a food composition table compiled by INSP [22]. We did not include nutrient intakes from supplement use in the calculation of total nutrient intake, and nutrients from fortification were reflected in the food composition tables. Daily intakes of essential nutrients derived from unprocessed red and processed meat were calculated, as well as total nutrient intake. The nutrients analyzed were: total energy intake, protein, fat, saturated fat, calcium, iron, nonheme iron, heme iron, magnesium, phosphorus, sodium, zinc, selenium, vitamin B5, riboflavin, niacin, vitamin B6, vitamin B12, and vitamin A. We also used the 24-h recall to disaggregate meat consumption into meat subcategories to understand how the Mexican population is consuming meat. These categories included the following: *1*) bacon, *2*) beef, *3*) ham, *4*) luncheon meat, *5*) organ meat, *6*) pork, *7*) sausage, and *8*) lamb/venison. We calculated the per capita mean daily consumption (g) of each meat food group and total meat consumption (g).

### Sociodemographic information

Sociodemographic variables were: age groups according to predetermined ENSANUT cutoffs (0–5, 6–11, 12–19, 20–59, and ≥60 y), sex (female compared with male), educational attainment of the head of household (none to middle school, high school, trade or professional training, and bachelors or higher), socioeconomic status based on a wealth index [23], area of residence (urban compared with rural), and indigenous status (whether the respondent reported speaking an indigenous language or not).

### Statistical analysis

We described the survey-weighted cross-sectional distribution of sociodemographic characteristics using “survey” package in R to account for the complex survey design and reported them by survey cycle.

We assessed trends in daily average consumption and in the proportion of participants reporting consumption of each food group (>0 g) over the 7-d recall for the FFQ using a survey-weighted generalized linear model with an “identity” link function to account for nonnormal distribution in consumption. Survey cycle was added to the model as a continuous variable and further adjusted for total energy intake to estimate the change in daily mean consumption (g) and mean prevalence of consumers (%) of each food group by survey cycle. We then calculated daily mean consumption (SE) and prevalence of consumers for each food by survey cycle. These estimates were obtained from the regression model holding total energy intake at the weighted sample mean across all survey cycles.

We then tested whether these trends varied by sociodemographic group. We ran multiple models with an interaction term of each sociodemographic variable with the continuous survey cycle in addition to adjusting for total energy intake. We reported daily mean consumption (SE) by survey cycle for each sociodemographic group using a regression model that adjusted for sociodemographic group and total energy intake, holding total energy intake at the mean for each level of the sociodemographic group. We also reported the average change in daily consumption [95% confidence interval (CI)] by survey cycle for each sociodemographic group through calculating the linear combination of regression coefficients from the regression models with interaction terms. *P* values were derived from a Wald test of the interaction term.

Finally, we performed a subgroup analysis where we evaluated trends among total meat consumers (≥0 g consumption of either unprocessed red or processed meat over the 7-d recall period) after adjusting for total energy intake.

### Nutrient contribution

With 24-h recall data, we summarized the survey-weighted average percent nutrient contribution (SE) to total nutrient intake for nutrients with contributions ≥10% from unprocessed red or processed meat. We additionally tested for differences in unprocessed red and processed meat contribution by sociodemographic groups using a Kruskal–Wallis test.

### Meat item contributions to meat intake

We determined survey-weighted population average percent contribution (SE) of each meat food subcategory to overall total meat consumption. We reported the contribution of meat food groups to total meat consumption for the general population. We then tested for differences by sociodemographic groups for meat food subcategory accounting for ≥10% of total meat consumption using a Kruskal–Wallis Test.

### Sensitivity analysis

Due to the smaller sample size in 2020 ENSANUT–Continua*,* we tested for trends excluding the 2020 cycle.

## Results

The most commonly consumed food groups were dairy and legumes: 96.4% (95% CI: 94.5%, 97.7%) and 94.8% (95% CI: 93.2%, 96.1%), respectively, of people in Mexico consumed these food groups in a given week in 2020. Most people also consumed some unprocessed red or processed meat (91.3%, 95% CI: 89.0%, 93.2%). There were significant declines in the prevalence of consumers in a given week of unprocessed red meat [mean 0.26 percentage points (pp)/survey cycle], seafood (mean 0.25 pp/survey cycle), and legumes (mean 0.19 pp/survey cycle) but a significant increase in the prevalence of poultry consumers (mean 0.31 pp/survey cycle) (Supplemental Figure 1).

**FIGURE 1:**
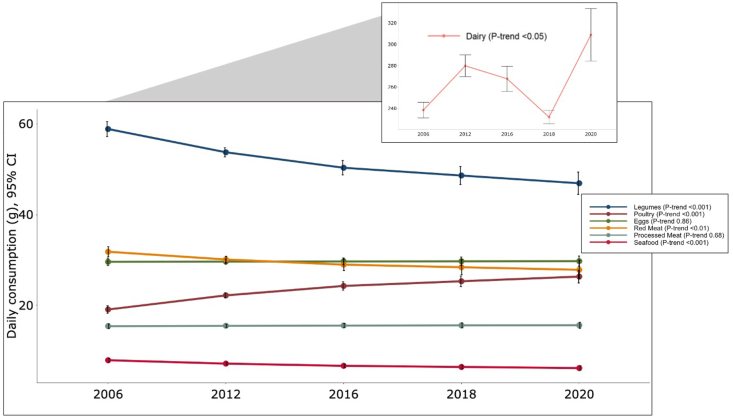
Trends in meat and other protein-rich food group intake among Mexican population of all ages, ENSANUT 2006–2020. Daily consumption (g) is predicted using survey-weighted generalized linear regression models, with survey cycle treated as a continuous variable and holding total energy intake at the mean 1814 kcal/d (±8.4) across all survey cycles. *P*-trend was obtained from a generalized linear model treating survey cycle as a continuous variable and adjusting for total energy intake. Legumes refer to “legumes, nuts, and seeds”; red meat refers to “unprocessed red meat.” CI, confidence interval; ENSANUT, Mexican National Health and Nutrition Survey.

Daily unprocessed red meat consumption declined by mean 0.28 g/survey cycle (*P*-trend < 0.01) over the period 2006 to 2020, whereas there was no overall change in daily processed meat consumption over time (*β* = 0.01, *P*-trend = 0.68) (Supplemental Table 2, Figure 1). Consumption of total meat (*β* = −0.27, *P*-trend < 0.05), seafood (*β* = −0.12 *P*-trend < 0.001), and legumes (*β* = −0.85, *P*-trend < 0.001) also declined over time. In contrast, poultry (*β* = 0.52, *P*-trend < 0.001) and dairy (*β* = 1.09, *P*-trend < 0.05) consumption increased.

Trends in unprocessed red and processed meat varied by sociodemographic group. Unprocessed red and processed meat consumption increased among those aged 6−19 y (*P*-interaction < 0.001), whereas it declined among those aged 60+y (TABLE 1, TABLE 2). Unprocessed red meat decreased in the urban population (mean 0.39 g/survey cycle) and did not change in the rural population. Additionally, unprocessed red and processed meat consumption declined by 0.84 g and 0.2 g, respectively, per survey cycle in the lowest income group but did not change in the middle- or highest-income groups. There were no differential trends in unprocessed red and processed meat consumption by educational attainment of the head of household. Processed meat remained unchanged in the urban population and increased in the rural population (mean 0.25 g/survey cycle).

**TABLE 1:** Trends in unprocessed red meat consumption (g per survey cycle) in Mexican population adjusted for total energy intake stratified by age group, sex, educational attainment of head of household, socioeconomic status, indigenous status, and area of residence, ENSANUT 2006–2020.

	2006 *n =* 38,775	2012 *n =* 6729	2016 *n =* 14,537	2018 *n =* 23,516	2020 *n =* 2033	Mean change per survey cycle (95% CI)^1^	*P* value^2^
Age group (y)							
0–5	10 (0.4)	10.1 (0.3)	10.1 (0.3)	10.2 (0.4)	10.2 (0.5)	0.01 (−0.1, 0.11)	<0.001^3^
6–11	17.9 (0.4)	18.7 (0.3)	19.2 (0.4)	19.5 (0.4)	19.8 (0.5)	0.14 (0.04, 0.23)	
12–19	31.6 (0.9)	33.3 (0.6)	34.4 (0.8)	34.9 (1)	35.5 (1.2)	0.28 (0.02, 0.53)	
20–59	39.6 (1.2)	37.8 (0.8)	36.6 (1.7)	36.1 (2.3)	35.5 (2.8)	−0.29 (−0.83, 0.25)	
60+	32.2 (1.3)	27.6 (0.7)	24.5 (1)	23 (1.2)	21.4 (1.5)	−0.77 (−1.1, −0.44)	
Sex							
Female	29.6 (0.5)	27.2 (0.4)	25.6 (0.5)	24.8 (0.6)	24 (0.7)	−0.4 (−0.54, −0.26)	0.23
Male	34.5 (1.1)	33.8 (0.6)	33.4 (1.4)	33.2 (1.8)	32.9 (2.3)	−0.11 (−0.56, 0.33)	
Educational attainment of HoH							
None to middle school	29.6 (0.6)	27.6 (0.4)	26.4 (0.8)	25.7 (1.1)	25.1 (1.3)	−0.32 (−0.57, −0.07)	
High school, trade or professional training	38.9 (1.5)	35.2 (0.9)	32.8 (0.9)	31.6 (1.1)	30.3 (1.3)	−0.61 (−0.93, −0.3)	0.24
Bachelor’s or higher	43.3 (1.9)	42.3 (1.2)	41.6 (1.7)	41.3 (2.1)	40.9 (2.6)	−0.17 (−0.7, 0.36)	
Socioeconomic status							
Low	29.4 (0.8)	24.3 (0.5)	21 (0.7)	19.3 (0.9)	17.6 (1.1)	−0.84 (−1.06, −0.62)	
Middle	28.9 (1.2)	27.7 (0.6)	26.8 (1.4)	26.4 (1.9)	26 (2.3)	−0.21 (−0.68, 0.26)	<0.001^3^
High	36.5 (0.9)	35.7 (0.6)	35.1 (1)	34.8 (1.3)	34.5 (1.6)	−0.14 (−0.44, 0.16)	
Indigenous							
No	32.4 (0.6)	30.7 (0.4)	29.5 (0.7)	29 (0.9)	28.4 (1.1)	−0.29 (−0.5, −0.08)	
Yes	21.6 (1.4)	19.5 (0.9)	18.1 (1)	17.5 (1.2)	16.8 (1.4)	−0.34 (−0.64, −0.04)	0.8
Area of residence							
Rural	21 (0.6)	21 (0.4)	21.1 (0.6)	21.1 (0.7)	21.1 (0.8)	0.01 (−0.16, 0.18)	
Urban	35.3 (0.7)	32.9 (0.4)	31.4 (0.8)	30.6 (1.1)	29.9 (1.3)	−0.39 (−0.64, −0.13)	<0.05^3^

Values are predicted using survey-weighted generalized linear regression models, with survey cycle treated as a continuous variable, and adjusting for sociodemographic group and total energy intake. Values represent survey-weighted mean grams consumed per capita (±SE) for each sociodemographic group, holding total energy intake within the sociodemographic group constant across all survey cycles.

Abbreviations: CI, confidence interval; ENSANUT, Mexican National Health and Nutrition Survey; HoH, head of house.

1 Average change per survey cycle per sociodemographic group is calculated as the linear combination of interaction terms derived from survey-weighted generalized linear regressions adjusted for survey year, sociodemographic factor, and total energy intake in addition to the interaction term. Interaction terms were sociodemographic factors multiplied by survey year to test for varying trends by sociodemographic factors.

2 *P* values were derived from a Wald test of interaction terms.

3 *P* values are significant at the *α* = 0.05 level.

**TABLE 2:** Trends in processed meat consumption (g per survey cycle) in Mexican population adjusted for total energy intake stratified by age group, sex, educational attainment of head of household, socioeconomic status, indigenous status, area of residence, ENSANUT 2006–2020.

	2006 *n =* 38,775	2012 *n =* 6729	2016 *n =* 14,537	2018 *n =* 23,516	2020 *n =* 2033	Mean change per survey cycle (95% CI)^1^	*P* value^2^
Age group (y)							
0–5	12.2 (0.5)	12.4 (0.3)	12.5 (0.3)	12.6 (0.4)	12.6 (0.5)	0.04 ( −0.07, 0.14)	
6–11	15.6 (0.4)	17 (0.3)	17.9 (0.3)	18.3 (0.4)	18.8 (0.5)	0.23 (0.13, 0.32)	
12–19	18 (0.4)	19.7 (0.3)	20.7 (0.4)	21.3 (0.4)	21.8 (0.5)	0.27 (0.16, 0.38)	<0.001^3^
20–59	17.2 (0.4)	15.7 (0.3)	14.7 (0.4)	14.2 (0.5)	13.8 (0.7)	−0.24 (−0.37, −0.11)	
60 +	10.5 (0.5)	9.3 (0.3)	8.4 (0.5)	8 (0.6)	7.5 (0.8)	−0.22 (−0.38, −0.05)	
Sex							
Female	14.1 (0.3)	14.1 (0.2)	14 (0.3)	14 (0.3)	14 (0.4)	−0.01 (−0.09, 0.07)	
Male	17 (0.4)	17.4 (0.2)	17.6 (0.4)	17.7 (0.4)	17.8 (0.5)	0.06 (−0.05, 0.17)	0.3
Educational attainment of HoH							
None to middle school	14.2 (0.3)	14.3 (0.2)	14.3 (0.3)	14.3 (0.3)	14.3 (0.4)	0.01 (−0.07, 0.08)	
High school, trade or professional training	19.5 (0.7)	19.2 (0.4)	19 (0.5)	18.9 (0.6)	18.8 (0.8)	−0.04 (−0.23, 0.14)	0.36
Bachelor’s or higher	20.6 (0.9)	19.7 (0.6)	19 (0.7)	18.7 (0.8)	18.4 (1)	−0.16 (−0.38, 0.06)	
Socioeconomic status							
Low	13.9 (0.5)	12.7 (0.3)	11.9 (0.5)	11.5 (0.6)	11.1 (0.7)	−0.2 (−0.35, −0.06)	
Middle	15.3 (0.4)	15.8 (0.3)	16.2 (0.3)	16.3 (0.4)	16.5 (0.5)	0.09 (−0.02, 0.2)	<0.01^3^
High	16.9 (0.4)	17.2 (0.2)	17.3 (0.3)	17.4 (0.4)	17.4 (0.5)	0.04 (−0.07, 0.14)	
Indigenous							
No	15.9 (0.3)	16 (0.2)	16 (0.2)	16 (0.3)	16 (0.4)	0.01 (−0.06, 0.08)	
Yes	6.8 (0.6)	6.9 (0.5)	6.9 (0.5)	7 (0.6)	7 (0.7)	0.01 (−0.12, 0.14)	1.0
Area of residence							
Rural	9.4 (0.4)	10.9 (0.2)	11.9 (0.3)	12.4 (0.3)	12.9 (0.4)	0.25 (0.16, 0.34)	
Urban	17.3 (0.3)	17 (0.2)	16.7 (0.3)	16.6 (0.3)	16.5 (0.4)	−0.06 (−0.15, 0.03)	<0.001^3^

Values are predicted using survey-weighted generalized linear regression models, with survey cycle treated as a continuous variable, and adjusting for sociodemographic group and total energy intake. Values represent survey-weighted mean grams consumed per capita (±SE) for each sociodemographic group, holding total energy intake within the sociodemographic group constant across all survey cycles.

Abbreviations: CI, confidence interval; ENSANUT, Mexican National Health and Nutrition Survey; HoH, head of house.

1 Average change per survey cycle per sociodemographic group is calculated as the linear combination of interaction terms derived from survey-weighted generalized linear regressions adjusted for survey year, sociodemographic factor, and total energy intake in addition to the interaction term. Interaction terms were sociodemographic factors multiplied by survey year to test for varying trends by sociodemographic factors.

2 *P* values were derived from a Wald test of interaction terms.

3 *P* values are significant at the *α* = 0.05 level.

Trends in seafood also varied by age-group: consumption increased over time in younger populations (6−11 y) and decreased in older populations (≥20 y) (Supplemental Table 3). Poultry consumption increased in those aged 0−59 y, and in the nonindigenous population (Supplemental Table 4). Dairy consumption increased in those aged 0−5 y, males, those living in rural areas, and those with middle socioeconomic status, whereas it decreased in older age groups (>20 y), and females (Supplemental Table 5). Egg consumption increased in those aged 0−19 y and in those with head of household that had either “high, trade, or professional training” or higher levels of education, and in the highest-income group, whereas it decreased in those aged 20−59 y, the lowest income group, those belonging to households whose head had “none to middle school education," and in indigenous participants (Supplemental Table 6). Legume consumption increased in younger ages (6−11 y) with reductions in older ages (>12 y) and greater reductions in rural compared with urban and in indigenous compared with nonindigenous groups (Supplemental Table 7).

Results were largely consistent when restricting to meat consumers (Supplemental Table 8). Among total meat consumers, average daily unprocessed red meat consumption was declining at 0.29 g/survey round (*P*-trend < 0.05) and processed meat had no significant changes over the period studied.

**FIGURE 2:**
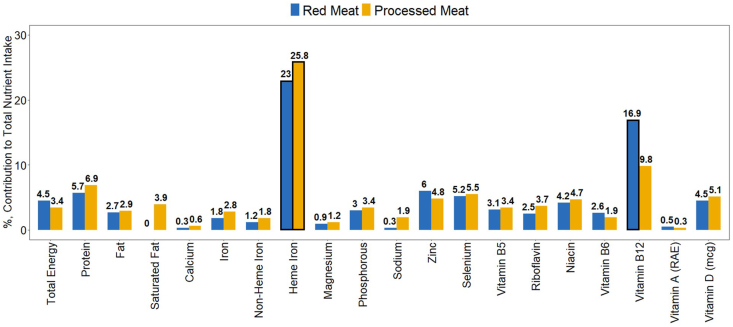
Survey-weighted unprocessed red and processed meat percent contribution to total nutrient intake per capita, all age groups, ENSANUT 2016. Total energy is defined as kcal/d. Values are percent contribution of unprocessed red and processed meat to total nutrient intake per capita. Bolded borders nutrient contributions ≥ 10%. Unweighted sample size for ENSANUT 2016 24-h dietary recall is *n =* 4070. ENSANUT, Mexican National Health and Nutrition Survey; RAE, Retinol Activity Equivalents.

Unprocessed red meat contributed, on average in 2016, to 23.0% of heme iron intake and 16.9% of vitamin B12 intake but <10% to all other nutrients evaluated in 2016 (Figure 2). Processed meat contributed, on average, to 25.8% of heme iron intake but <10% of all other nutrients evaluated in 2016. Unprocessed red meat accounted for a greater proportion of heme iron and vitamin B12 intake among older ages than younger, whereas processed meat contributed more among younger ages than older (Supplemental Table 9). Unprocessed red and processed meat contributed significantly less heme iron and vitamin B12 to indigenous compared with nonindigenous participants. Processed meat contributed more heme iron and vitamin B12 to those with higher than lower socioeconomic status but no differences were observed for unprocessed red meat by socioeconomic status. Processed meat contributed more heme iron among those with higher educational attainment of heads of households than lower but not vitamin B12 and no differences were found for unprocessed red meat. Lastly, no differences were observed for nutrient contribution by sex.

**FIGURE 3:**
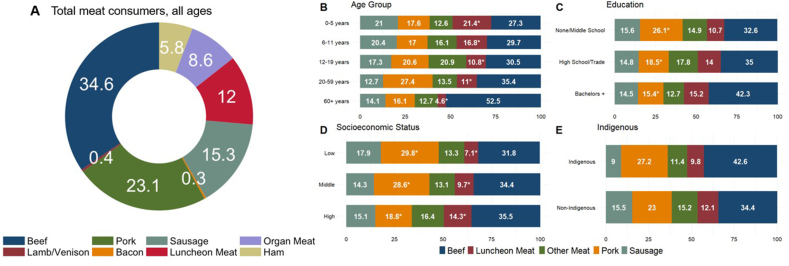
Survey-weighted meat subcategory percent contribution to total meat consumption among total meat consumers, all age groups, ENSANUT 2016. Values are meat subcategories percent contribution to total meat intake among total meat consumers. Unweighted sample size of total meat consumers in ENSANUT 2016 24-h dietary recall is *n =* 2280 out of sample of *n =* 4070. Meat subcategories accounting for < 10% of total meat intake among total meat consumers in Mexican population, all ages. Lamb/venison, organ meat, and bacon were consolidated into “Other Meat” for sociodemographic analyses. Education in panel c refers to educational attainment of the head of household. ∗Indicates statistically significant differences by sociodemographic characteristic at the *α* = 0.05 obtained through the Kruskal–Wallis test for complex survey samples. ENSANUT, Mexican National Health and Nutrition Survey.

The meat subcategories with the largest contributions to total meat consumption among total meat consumers in 2016 were beef (34.6%), pork (23.1%), sausage (15.3%), and luncheon meat (12.0%) (Figure 3A). Luncheon meat contributed a higher proportion of total meat among younger ages than older (Figure 3B). Pork comprised more of total meat among those with higher levels of educational attainment of the head of household compared with lower (Figure 3C). Luncheon meat represented a greater proportion of total meat among the highest-income group compared with the lowest whereas pork represented a larger proportion of total meat among the lowest income group compared with the highest (Figure 3D). Meat subcategory contribution to total meat consumption did not differ by indigenous status (Figure 3E).

In the sensitivity analysis where we excluded the 2020 cycle, trends during 2006 to 2018 varied from those observed during 2006 to 2020 (Supplemental Table 10). Unprocessed red meat consumption was stable (*P*-trend = 0.82), whereas processed meat consumption increased, 0.28 g/survey cycle (*P*-trend < 0.001), and so did total meat (*β* = 0.23; *P*-trend < 0.001) from 2006 to 2018. In terms of other food groups, seafood marginally declined (*β* = −0.07; *P*-trend < 0.01) and legumes declined (*β* = −0.82; *P*-trend < 0.01). Poultry and eggs increased (*β* = 0.41; *P*-trend < 0.01 and (*β* = 0.13; *P*-trend = 0.01, respectively). Dairy consumption was stable during 2006–2018 (*P*-trend = 0.39).

## Discussion

We found that mean daily consumption of unprocessed red meat experienced slight but statistically significant declines — <1 g, on average, per survey cycle — in the Mexican population from 2006 to 2020, whereas processed meat remained stable overall with a moderate upward trend in young people. Processed meats such as sausage or luncheon meat also accounted for a significantly larger proportion of total meat consumption among 0–5 and 6–11 y age groups compared with older age groups. Average per capita consumption of seafood and legumes went down whereas poultry and dairy went up. The magnitude of these changes is unlikely to have significant nutritional health impacts; however, they indicate that adherence to the 2023 Mexican Dietary Guidelines is not improving with time, at least in the short- and medium-term if current trends continue. Moreover, consumption is above 2023 Mexican Dietary Targets (36 g/d compared with a target of 30 g/d for unprocessed red meat in the adult population (20 y+) and 7 g/d compared with 0 g/d of processed meat in the adult population in 2020), and at current levels, poses a dietary risk factor for several chronic diseases and negative environmental impacts [7].

We also found that unprocessed red and processed meat importantly contribute to heme iron and vitamin B12 intake but observed trends are unlikely to affect short-run intake of these nutrients [7]. Given that these findings were based on the 2016 dietary recall, they may not be representative of nutrient contribution over time given potential changes other factors such as food composition or fortification. Furthermore, when we evaluated trends in consumption excluding 2020, we observed that unprocessed meat was constant and that processed and total meat were increasing as opposed to stable and declining, respectively. Therefore, the slight reductions in unprocessed meat from 2006 to 2020 may point to stable trends. And as we found that processed meat was stable from 2006 to 2020 but increasing over 2006–2018, its consumption may be stabilizing as well. This reinforces the need for government-supported interventions that accelerate reductions in unprocessed red and processed meat and increase consumption of alternatives such as legumes and seafood to meet sustainable and healthy dietary targets set by the Dietary Guidelines.

Our findings suggest unprocessed red meat consumption is gradually declining, whereas processed meat remains stable. This contrasts longer term trends, where from 1984 to 2016, there were modest declines in unprocessed red meat purchases in Mexico (−26.5%) but large increases (+83.3%) in processed meat and ultraprocessed meat purchases (i.e., sausages) (+225.1%) [9]. However, more recent food purchases from 2006 to 2016 align with the findings of our study [9]. We hypothesize that the stabilization of trends—particularly for processed meat—is likely due to a settling effect post the enactment of the North American Free Trade agreement in 1994. The policy brought substantial changes to the Mexican diet, such as an increase in processed and ultraprocessed foods, which were uncommonly consumed prior [24]. Mexico may now be in an advanced stage of the nutrition transition compared with earlier periods [25]. As a result, meat consumption may have stabilized, although it remains high. Moreover, when we excluded 2020, unprocessed red meat was stable, whereas processed meat showed an upward trend. This may point to changes in eating patterns during the COVID-19 pandemic, such as purchasing more prepared foods and heightened food insecurity, which would have influenced trends over the full study period [26,27]. Purchases of foods such as nonbasic energy-dense foods and sugar-sweetened beverages also increased in 2020 compared with 2018, so a greater share of the household budget could have been allocated to these foods instead of meat—although increases in processed meat purchases were observed in the lowest income quintile in 2020 [27]. Discrepancies in trends between our findings and those derived from food purchases could be attributed to the nature of food purchase data, which reflects household-level food acquisition not individual-level consumption that our results may better represent [28]. Moreover, household food expenditure surveys often exclude foods consumed away from home and, as eating away from home has increased in Mexico over recent decades [29], relying on food purchases could further bias the evaluation of temporal trends.

Our findings in the Mexican population are similar to those reported in the United States. An analysis using nationally representative dietary data evaluated trends in the United States adult population (≥20 y) from 1999 to 2016 and similarly found that red meat declined, whereas poultry increased, and there were no changes in processed meat and seafood consumption [30]. This reinforces the idea that Mexico, similar to the United States, is in later stages of the nutrition transition.

There are following 2 key takeaways from varying trends by sociodemographic groups: *1*) high levels coupled with increases in processed meat consumption in younger age groups may inform future population disease burden, and *2*) diets in rural populations may be becoming more similar to urban diets. Younger age groups tend to develop their dietary preferences during childhood [31], and these dietary habits may track to adulthood [32]. Therefore, these cohorts may increase the length of their exposure to “unhealthy” diets but also childhood eating habits have been associated with health outcomes onset in adulthood [33]. For example, higher adherence to “Western” dietary patterns (high in sweet and salty foods, refined grains, and unprocessed red and processed meat) in adolescence was associated with precursors to rectal and colorectal cancer and type 2 diabetes in adult middle-aged females in the United States [[34], [35]]. Processed meat was notably increasing in younger ages, with “luncheon meat” constituting a significantly larger proportion of total meat consumption in this group in 2016. Although observed increases were small, evidence indicates that no amount of processed meat is “safe” without increasing disease risk [36] and it has been classified as group I carcinogen [2,21]. Thus, monitoring its trends and reducing both the duration and dose of exposure is a public health priority. Next, we observed increases in meat and dairy together with declines in legumes in rural populations which may signal shifting dietary patterns in this group. Previous evaluations have found that rural populations tend to eat higher quality diets closer to the traditional Mexican diet (high in beans, whole grains, seafood, and lower in meat) [11,37]. Whereas, urban populations consumed “Westernized” diets high in animal-sourced foods [8,11,25,38]. Therefore, rural diets may have lagged in this dietary transition and be catching up to urban diets over time.

With respect to other food groups, we found that overall seafood and legumes declined over time, whereas dairy and poultry increased. This is reflected in findings from other studies. Substantial increases in household food purchases of poultry with modest increases in seafood and reductions in legumes were observed from 1984 to 2016 [9]. These longer term trends are consistent with ours, likely because these foods were not newly introduced to the Mexican diet like processed meat. These trends are important as they offer more sustainable and healthier alternatives to unprocessed red and processed meat. However, adherence to dietary targets for these foods remains low in Mexico, especially for seafood and legumes, which our findings suggest is unlikely to improve in the short term [10,11]. Only 1.4% of children aged 6–11 y, 0.2% of adolescents aged 12–19, and 1.0% of adults 20+ y are meeting the guideline recommendations and adherence to the seafood recommendation is lower among younger age groups than older indicating that substantial increases are needed [10].

Nutrients of concern in Mexico are vitamin B12 and iron, especially for lower income and indigenous groups [11,39]. We found that, in 2016, unprocessed red meat importantly contributed to heme iron and vitamin B12, but that processed meat was only important for heme iron. Thus, efforts should prioritize reducing processed meat consumption, particularly among young people. Such reductions are unlikely to significantly impact nutrients of concern, especially if substituted for healthier alternatives like beans, eggs, poultry, or fish as recommended by the Dietary Guidelines [7]. Therefore, further policy intervention should discourage consumption of processed meat while simultaneously emphasizing healthier, more sustainable dietary substitutions to ensure improvements in diet quality.

To our knowledge, this is the first study to evaluate recent nationally representative trends in unprocessed red and processed meat consumption in Mexico. However, self-reported diet measurement tools such as the FFQ and 24-h dietary recall are susceptible to measurement error [19] and reporting of dietary intake by parents/caretakers for children aged <12 y may have introduced bias such as underreporting [40]. Moreover, the 2006 FFQ included 101 items that were incorporated into the 140-item version used from 2012 onwards. However, we ensured comparability of the food items categorized into the food groups analyzed. Additionally, FFQs tend to more accurately capture usual consumption so would have captured these food groups—with the caveat that seasonal variation of foods consumed may not be captured beyond the recall period—whereas 24-h dietary recalls more accurately capture average population-level consumption [19]. Although measurement error may influence absolute intake at a given time, it is unlikely to influence trends because the error will likely remain unchanged over time. Furthermore, adjustment for energy intake helps reduce bias and enhance comparability across time [19]. Another potential source of bias could be if the FFQ does not reflect new meat-containing foods, foods away from home, or increases in meat portions within mixed dishes which may be differential over time and across sociodemographic groups. Lastly, we did observe differential results in trends when 2020 was excluded. However, although the sample size is smaller, 2020 ENSANUT was designed to be comparable to preceding cycles and is nationally representative for which we accounted for the complex survey design in our statistical analyses [17]. Although the variance may have been wider due to smaller sample size, incorporating the survey design and weights produces comparable point estimates. Rather, the differences observed are likely due to shifting trends in consumption such as from a stable to downward trend in unprocessed meat consumption and an upward to stable trend in processed meat consumption. Nevertheless, the key finding is consistent; unprocessed red and processed meat are not declining at the rate needed to meet healthy and sustainable dietary targets. Future studies should add additional years of dietary information to further examine the trajectory of consumption trends. Lastly, it would be interesting to evaluate whether the types of unprocessed red and processed meat being consumed vary over time and by subgroup.

Trends in the consumption of unprocessed red meat and processed meat have the potential to impact the environment, nutritional status, and disease outcomes. Overall, we found that unprocessed red meat consumption may be stable, with slight decreases observed from 2006 to 2020, whereas processed meat consumption remains high and unchanged. There were simultaneous increases in poultry and dairy but declines in legumes and seafood. Further research is needed to understand the dietary substitution behaviors in the food groups with changing trends in consumption. Given that processed meat consumption has incrementally increased in younger age groups, that it is not an important source of key micronutrients, and that it is associated with negative health consequences particularly colorectal cancer, researchers, and policymakers should prioritize enhancing existing policies in Mexico such as front-of-pack labeling, food-specific taxes, and regulations of school-based food environments [41] targeting younger age groups.

## Author contributions

The authors’ responsibilities were as follows – KC: designed the study with guidance from LMJ, PA, JAR, CB: collated and analyzed the data, and interpreted the results and wrote the manuscript, and had primary responsibility for final content; LMJ, PA, JAR, CB: provided substantial comments to the interpretation of results and manuscript draft; and all authors: read and approved the final version of the manuscript.

## Ethical statement

This study was conducted according to the guidelines laid down in the Declaration of Helsinki and all procedures involving research study participants for Mexican National Health and Nutrition Survey (ENSANUT) 2006 to ENSANUT 2020 were approved by the Committee for Research, Ethics, Biosecurity at the National Institute of Public Health (INSO), Cuernavaca, Mexico. Written informed consent was obtained from all subjects/patients.

## Data availability

The national diet data are available through the ENSANUT https://ensanut.insp.mx/.

## Funding

KC received financial support from the Edinburgh Earth, Ecology, and Environment Doctoral Training Partnership (E4 DTP). PA and LMJ acknowledge UK Research and Innovation support through BB/W018152/1 and Wellcome through 227154/Z/23/Z. The funders had no role in the study design, data collection, analysis, interpretation of the data, nor writing or decision to submit this manuscript.

## Conflict of interest

The authors declare no conflict of interest. For the purpose of open access, the author has applied a Creative Commons Attribution (CC BY) license to any Author Accepted Manuscript version arising from this submission.

## References

[1] Crippa M., Solazzo E., Guizzardi D., Monforti-Ferrario F., Tubiello F.N., Leip A. (2021). Food systems are responsible for a third of global anthropogenic GHG emissions. Nat. Food..

[2] Bouvard V., Loomis D., Guyton K.Z., Grosse Y., Ghissassi F.E., Benbrahim-Tallaa L. (2015). Carcinogenicity of consumption of red and processed meat. Lancet Oncol.

[3] Rouhani M.H., Salehi-Abargouei A., Surkan P.J., Azadbakht L. (2014). Is there a relationship between red or processed meat intake and obesity? A systematic review and meta-analysis of observational studies: red or processed meat and obesity. Obes. Rev..

[4] Gu X., Drouin-Chartier J.-P., Sacks F.M., Hu F.B., Rosner B., Willett W.C. (2023). Red meat intake and risk of type 2 diabetes in a prospective cohort study of United States females and males. Am. J. Clin. Nutr..

[5] Abete I., Romaguera D., Vieira A.R., de Munain A.L., Norat T. (2014). Association between total, processed, red and white meat consumption and all-cause, CVD and IHD mortality: a meta-analysis of cohort studies. Br. J. Nutr..

[6] Chen G.-C., Lv D.-B., Pang Z., Liu Q.-F. (2013). Red and processed meat consumption and risk of stroke: a meta-analysis of prospective cohort studies. Eur. J. Clin. Nutr..

[7] Secretaría de Salud (SSA), Instituto Nacional de Salud Pública (INSP), & Grupo Intersectorial de Salud, Alimentación, Medio Ambiente y Competitividad (GISAMAC). (2022). Guías alimentarias saludables y sostenibles para la población mexicana. México: Secretaría de Salud. Retrieved from: https://www.gob.mx/salud.

[8] Rivera J.A., Barquera S., González-Cossío T., Olaiz G., Sepúlveda J. (2004). Nutrition transition in Mexico and in other latin American countries. Nutr. Rev..

[9] Marrón-Ponce J.A., Tolentino-Mayo L., Hernández-F M., Batis C. (2018). Trends in ultra-processed food purchases from 1984 to 2016 in Mexican households. Nutrients.

[10] Martinez-Tapia B., Rodríguez-Ramírez S., Valenzuela-Bravo D.G., Medina-Zacarías M.C., Gaona-Pineda E.B., Arango-Angarita A. (2025).

[11] Castellanos-Gutiérrez A., Sánchez-Pimienta T.G., Batis C., Willett W., Rivera J.A. (2021). Toward a healthy and sustainable diet in Mexico: where are we and how can we move forward?. Am. J. Clin. Nutr..

[12] Frank S.M., Jaacks L.M., Batis C., Vanderlee L., Taillie L.S. (2021). Patterns of red and processed meat consumption across North America: a nationally representative cross-sectional comparison of dietary recalls from Canada, Mexico, and the United States. Int. J. Environ. Res. Public Health..

[13] Aburto T.C., Batis C., Pedroza-Tobías A., Pedraza L.S., Ramírez-Silva I., Rivera J.A. (2022). Dietary intake of the Mexican population: comparing food group contribution to recommendations, 2012-2016. Salud Publica Mex.

[14] Reyes-Garcia A., Stern D., Rivera-Dommarco J., Batis C. (2022). Changes in food intake from 1999 to 2012 among Mexican children and women. Br. J. Nutr..

[15] (2019). Resultados Nacionales, 2018-2019.

[16] Shamah-Levy T., Romero-Martínez M., Barrientos-Gutiérrez T., Cuevas-Nasu L., Bautista-Arredondo S., Colchero M.A. (2022). Encuesta Nacional de Salud y Nutrición 2021 sobre Covid-19: Resultados nacionales.

[17] Shamah-Levy T., Romero-Martínez M., Barrientos-Gutierrez T., Cuevas-Nasu L., Bautista-Arredondo S., Colchero M.A. (2021).

[18] Ramírez-Silva I., Jiménez-Aguilar A., Valenzuela-Bravo D., Martinez-Tapia B., Rodríguez-Ramírez S., Gaona-Pineda E.B. (2016). Methodology for estimating dietary data from the semi-quantitative food frequency questionnaire of the Mexican National Health and Nutrition Survey 2012. Salud Publica Mex.

[19] Willet W. (2012).

[20] Denova-Gutiérrez E., Ramírez-Silva I., Rodríguez-Ramírez S., Jiménez-Aguilar A., Shamah-Levy T., Rivera-Dommarco J.A. (2016). Validity of a food frequency questionnaire to assess food intake in Mexican adolescent and adult population. Salud Pública México.

[21] International Agency for Research on Cancer (IARC) (2018). Red meat and processed meat (IARC. Monographs on the Evaluation of Carcinogenic Risks to Humans.

[22] Instituto Nacional de Salud Pública (INSP) (2012). Base de datos del valor nutritivo de los alimentos [Database of food’s nutritional value].

[23] Batis C., Aburto T.C., Sánchez-Pimienta T.G., Pedraza L.S., Rivera J.A. (2016). Adherence to dietary recommendations for food group intakes is low in the Mexican population. J Nutr.

[24] Clark S.E., Hawkes C., Murphy S.M.E., Hansen-Kuhn K.A., Wallinga D. (2012). Exporting obesity: US farm and trade policy and the transformation of the Mexican consumer food environment. Int. J. Occup. Environ. Health..

[25] Popkin B.M., Reardon T. (2018). Obesity and the food system transformation in Latin America. Obes. Rev. Off. J. Int. Assoc. Study Obes..

[26] Gaitán-Rossi P., Vilar-Compte M., Teruel G., Pérez-Escamilla R. (2021). Food insecurity measurement and prevalence estimates during the COVID-19 pandemic in a repeated cross-sectional survey in Mexico. Public Health Nutr.

[27] Sánchez-Ortiz N.A., Colchero M.A. (2023). Changes in food and beverage purchases associated with the coronavirus disease pandemic in Mexico – PubMed. J. Acad. Nutr. Diet..

[28] Smith, L. C., Dupriez, O., & Troubat, N. (2014 Feb). Assessment of the reliability and relevance of the food data collected in national household consumption and expenditure surveys (IHSN Working Paper No. 008). Washington, DC: International Household Survey Network.

[29] García-Chávez C.G., Barrientos-Gutierrez T., Ng S.W., Rivera J.A., Colchero M.A. (2025). Changes in sugar-sweetened beverages and non-essential energy-dense food purchases overall and by type before and after the implementation of taxes in Mexico: repeated cross-sectional national surveys (2008–2018). BMJ Public Health.

[30] Zeng L., Ruan M., Liu J., Wilde P., Naumova E.N., Mozaffarian D. (2019). Trends in processed meat, unprocessed red meat, poultry, and fish consumption in the United States, 1999-2016. J. Acad. Nutr. Diet..

[31] Venter C., Harris G. (2009). The development of childhood dietary preferences and their implications for later adult health. Nutr. Bull..

[32] Mikkilä V., Räsänen L., Raitakari O.T., Pietinen P., Viikari J. (2005). Consistent dietary patterns identified from childhood to adulthood: the cardiovascular risk in Young Finns Study. Br. J. Nutr..

[33] Jääskeläinen P., Magnussen C.G., Pahkala K., Mikkilä V., Kähönen M., Sabin M.A. (2012). Childhood nutrition in predicting metabolic syndrome in adults: the cardiovascular risk in young finns study. Diabetes Care.

[34] Malik V.S., Fung T.T., van Dam R.M., Rimm E.B., Rosner B., Hu F.B. (2012). Dietary patterns during adolescence and risk of type 2 diabetes in middle-aged women. Diabetes Care.

[35] Nimptsch K., Malik V.S., Fung T.T., Pischon T., Hu F.B., Willett W.C. (2014). Dietary patterns during high school and risk of colorectal adenoma in a cohort of middle-aged women. Int. J. Cancer..

[36] Haile D., Harding K.L., McLaughlin S.A., Ashbaugh C., Garcia V., Gilbertson N.M. (2025). Health effects associated with consumption of processed meat, sugar-sweetened beverages and trans fatty acids: a burden of proof study. Nat. Med..

[37] Pérez-Tepayo S., Rodríguez-Ramírez S., Unar-Munguía M., Shamah-Levy T. (2020). Trends in the dietary patterns of Mexican adults by sociodemographic characteristics. Nutr. J..

[38] Gaona-Pineda E.B., Martínez-Tapia B., Arango-Angarita A., Valenzuela-Bravo D., Gómez-Acosta L.M., Shamah-Levy T. (2018). Consumo de grupos de alimentos y factores sociodemográficos en población mexicana. Salud Pública México.

[39] Rivera J.A., Pedraza L.S., Aburto T.C., Batis C., Sánchez-Pimienta T.G., González de Cosío T. (2016). Overview of the dietary intakes of the Mexican population: results from the National Health and Nutrition Survey 2012. J. Nutr..

[40] Weden M.M., Brownell P.B., Rendall M.S., Lau C., Fernandes M., Nazarov Z. (2013). Parent-reported height and weight as sources of bias in survey estimates of childhood obesity. Am. J. Epidemiol..

[41] Rivera J.A., Colchero M.A., Pérez-Ferrer C., Barquera S. (2024). Perspective: Mexico’s experience in building a roolkit for obesity and noncommunicable diseases prevention. Adv. Nutr..

