# Supplementary material for: Trends in Unprocessed Red and Processed Meat Consumption in the Mexican Population, 2006–2020
Source: Curr Dev Nutr. 2025 Nov 12;9(12):107598. doi: 10.1016/j.cdnut.2025.107598 (PMC12718140; doi:10.1016/j.cdnut.2025.107598)
Supplement: Multimedia component 1 [file mmc1.docx]

**Supplementary Materials for**

**Trends in unprocessed red and processed meat consumption in the Mexican population, 2006-2020**

Kaela Connors,^1,2*^, Lindsay M. Jaacks^2^, Peter Alexander ^1,2^, Juan A. Rivera^3^, Carolina Batis^4^

^1^ School of Geosciences, University of Edinburgh, Drummond Street, Edinburgh, UK

^2^ Global Academy of Agriculture and Food Systems, University of Edinburgh, Midlothian, UK

^3^ Center for Health Systems Research, National Institute of Public Health, Cuernavaca, Morelos, Mexico

^4^ Center for Nutrition and Health Research, National Institute of Public Health, Cuernavaca, Morelos, Mexico

**Corresponding author:**

Kaela Connors

School of Geosciences

Global Academy of Agriculture and Food Systems

University of Edinburgh

Edinburgh, UK

Telephone: +44 0131 650 6586

Email: [k.m.connors@sms.ed.ac.uk](about:blank)

Contents

[Supplemental Table 1 – Characteristics of Mexican population, all ages, ENSANUT 2006 - 2020 3](#_Toc181344021)

[Supplemental Figure 1 – Prevalence of consumers over the past week of food groups by survey-cycle in Mexican Population, all ages, ENSANUT 2006 – 2020 4](#_Toc181344022)

[Supplemental Table 2 – Trends in consumption of meat and other protein-rich foods among the Mexican population, all ages, ENSANUT 2006 – 2020 5](#_Toc181344023)

[Supplemental Table 3 – Trends in seafood consumption in Mexican population stratified by age group, sex, educational attainment, socioeconomic status, indigenous status, area of residence, ENSANUT 2006 - 2020 6](#_Toc181344024)

[Supplemental Table 4 – Trends in poultry consumption in Mexican population by age group, sex, educational attainment, socioeconomic status, indigenous status, area of residence, ENSANUT 2006 - 2020 7](#_Toc181344025)

[Supplemental Table 5 – Trends in dairy consumption in Mexican population by age group, sex, educational attainment, socioeconomic status, indigenous status, area of residence, ENSANUT 2006 - 2020 8](#_Toc181344026)

[Supplemental Table 6 – Trends in egg consumption in Mexican population stratified by age group, sex, educational attainment, socioeconomic status, indigenous status, area of residence, ENSANUT 2006 - 2020 9](#_Toc181344027)

[Supplemental Table 7 – Trends in legumes, nuts seeds consumption in Mexican population by age group, sex, educational attainment, socioeconomic status, indigenous status, area of residence, ENSANUT 2006 - 2020 10](#_Toc181344028)

[Supplementary Table 9 - Survey-weighted red and processed meat percent contribution to total nutrient intake per capita by age group, ENSANUT 2016 12](#_Toc181344029)

[Supplementary Table 9 (cont) - Survey-weighted red and processed meat percent contribution to total nutrient intake per capita by sex and indigenous status, ENSANUT 2016 13](#_Toc181344030)

[Supplementary Table 9 (cont.) - Survey-weighted red and processed meat percent contribution to total nutrient intake per capita by socioeconomic status, all ages, ENSANUT 2016 14](#_Toc181344031)

[Supplementary Table 9 (cont) - Survey-weighted red and processed meat percent contribution to total nutrient intake per capita by educational attainment, all age groups, ENSANUT 2016 15](#_Toc181344032)

# **Supplemental Table 1 – Characteristics of Mexican population, all ages, ENSANUT 2006 - 2020**

|  | **2006**  **n = 38,775** | **2012**  **n = 6,729** | **2016**  **n = 14,537** | **2018**  **n = 23,516** | **2020**  **n = 2,033** |
| --- | --- | --- | --- | --- | --- |
| **Age group** |  |  |  |  |  |
| 0-5 years | 7.4 (7.11, 7.8) | 7.8 (7.1, 8.5) | 8.4 (7.8, 9.1) | 9.0 (8.5, 9.5) | 14.9 (13.3, 16.6) |
| 6-11 years | 16.6 (16.1, 17.1) | 13.9 (13.0, 15.0) | 16.0 (14.8, 17.2) | 17.6 (16.9, 18.3) | 25.4 (23.0, 27.9) |
| 12-19 years | 17.9 (17.5, 18.4) | 17.0 (15.8, 18.3) | 16.3 (15.5, 17.1) | 20.1 (19.3, 20.8) | 28.7 (25.8, 31.6) |
| 20-59 years | 46.9 (46.0, 47.7) | 48.5 (46.6, 50.5) | 47.9 (46.4, 49.5) | 32.1 (31.0, 33.2) | 22.2 (18.3, 26.8) |
| ≥ 60 years | 11.2 (10.4, 11.9) | 12.8 (11.3, 14.5) | 11.4 (10.2, 12.7) | 21.3 (20.0, 22.5) | 8.8 (6.6, 11.7) |
| **Sex** |  |  |  |  |  |
| Female | 56.5 (55.6, 57.4) | 51.0 (49.1, 52.8) | 52.3 (50.8, 53.7) | 67.1 (66.3, 68.0) | 51.1 (47.6, 54.6) |
| **Speaks Indigenous Language** | 6.3 (5.3, 7.6) | 3.4 (2.7, 4.1) | 5.6 (4.2, 7.4) | 5.5 (4.4, 6.8) | 3.1 (1.89, 5.02) |
| **Educational Attainment of head of household** |  |  |  |  |  |
| None to Middle School | 79.5 (78.1, 80.9) | 76.4 (74.6, 78.2%) | 72.6 (69.7, 75.3) | 70.0 (68.2, 71.7) | 71.2 (67.5, 74.6) |
| High School, trade, or professional training | 12.6 (11.8, 13.5) | 16.2 (14.7, 17.7) | 17.8 (16.2, 19.5) | 18.4 (17.2, 19.7) | 18.3 (15.7, 21.3) |
| Bachelor’s or higher | 7.9 (6.92, 8.94) | 7.4 (6.4, 8.5) | 9.6 (7.81, 11.8) | 11.6 (10.3, 13.0) | 10.5 (8.53, 12.9) |
| **Wealth Index** |  |  |  |  |  |
| Low | 25.9 (24.3, 27.6) | 37.4 (35.1, 39.8) | 16.0 (14.0, 18.2) | 25.0 (23.0, 27.1) | 26.7 (22.3, 31.5) |
| Middle | 29.3 (28.0, 30.6) | 30.3 (28.4, 32.2) | 32.3 (29.9, 34.8) | 33.4 (31.4, 35.5) | 25.7 (22.5, 29.1) |
| High | 44.8 (42.9, 46.7) | 32.3 (30.3, 34.4) | 51.7 (48.4, 54.9) | 41.6 (38.9, 44.3) | 47.6 (42.8, 52.5) |
| **Urban residence** | 77.0 (75.2, 78.7) | 74.9 (72.7, 77.1) | 74.3 (71.0, 77.3) | 76.6 (74.2, 78.9) | 81.8 (78.1, 85.0) |

Values are a survey-weighted proportion % (95% CI) unless otherwise specified.

# **Supplemental Figure 1 – Prevalence of consumers over the past week of food groups by survey-cycle in Mexican Population, all ages, ENSANUT 2006 – 2020**


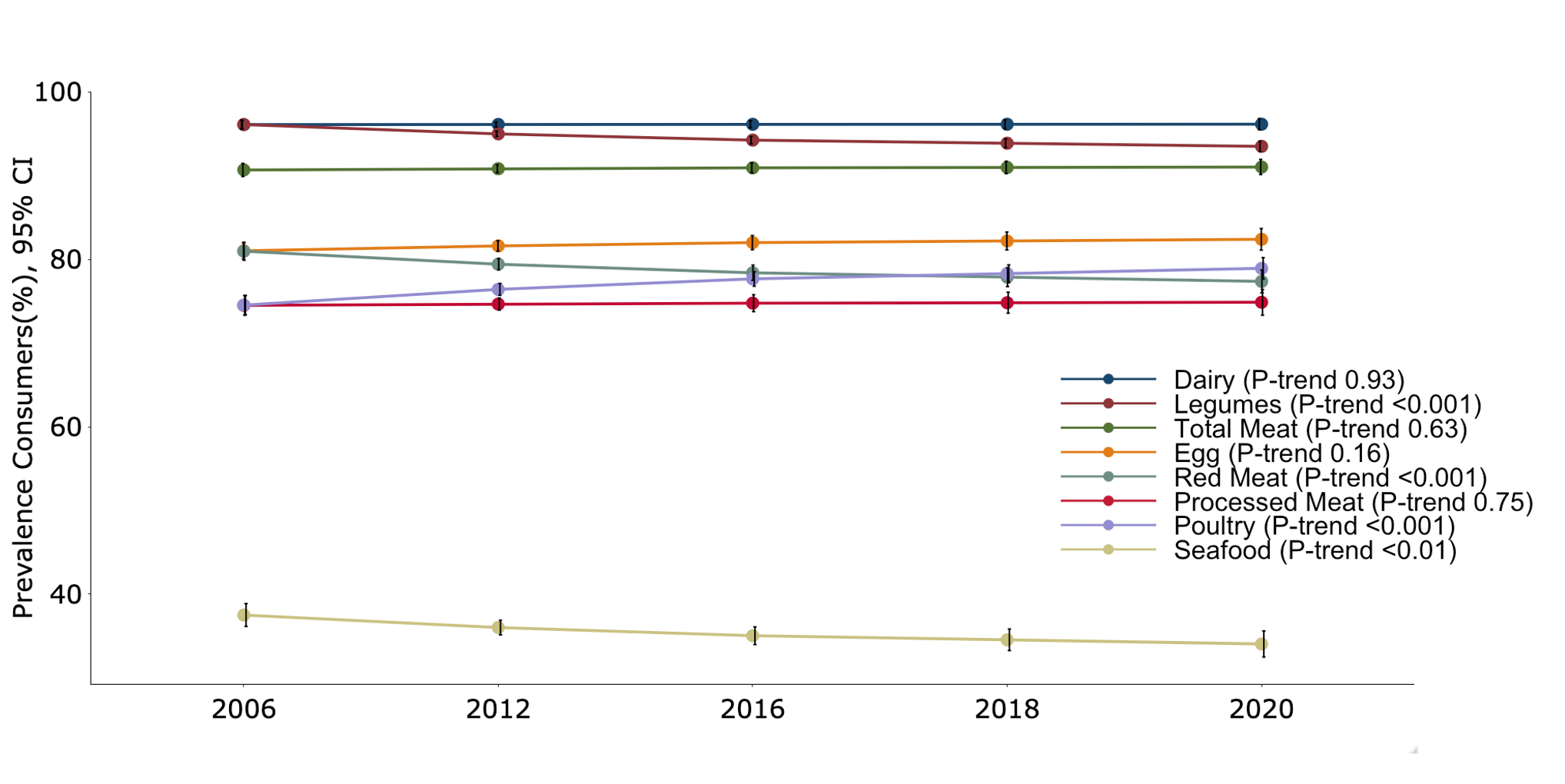


Trends are predicted survey-weighted percentage point change in prevalence of consumers for each food group for seven-day recall (%) (95% CI), holding total energy intake at the average 1,814 kcal/day (±8.4) across all survey cycles. P-trends were obtained from survey-weighted generalized linear regression including each survey-cycle as a continuous variable and adjusting for total energy intake.

Legumes correspond to ‘legumes, nuts, and seeds’; Total Meat corresponds to the sum of unprocessed red meat and processed meat; Red meat corresponds to unprocessed red meat

# **Supplemental Table 2 – Trends in consumption of meat and other protein-rich foods among the Mexican population, all ages, ENSANUT 2006 – 2020**

|  | **2006**  **n = 38,775** | **2012**  **n = 6,729** | **2016**  **n = 14,537** | **2018**  **n = 23,516** | **2020**  **n = 2,033** | **Average change per survey cycle**  **(95% CI) **** | **P-trend†** |
| --- | --- | --- | --- | --- | --- | --- | --- |
| Unprocessed  Red Meat | 31.85 (0.56) | 30.14 (0.36) | 29 (0.66) | 28.43 (0.85) | 27.86 (1.04) | **-0.28 (-0.49, -0.08)** | **<0.01** |
| Processed Meat | 15.44 (0.26) | 15.53 (0.16) | 15.59 (0.23) | 15.62 (0.28) | 15.65 (0.34) | 0.01 (-0.06, 0.09) | 0.68 |
| Total Meat* | 47.28 (0.68) | 45.66 (0.43) | 44.58 (0.75) | 44.04 (0.96) | 43.51 (1.18) | **-0.27 (-0.50, -0.04)** | **<0.05** |
| Seafood | 7.93 (0.22) | 7.19 (0.14) | 6.69 (0.18) | 6.45 (0.21) | 6.2 (0.26) | **-0.12 (-0.18, -0.07)** | **<0.001** |
| Poultry | 19.12 (0.42) | 22.23 (0.28) | 24.3 (0.47) | 25.34 (0.59) | 26.38 (0.72) | **0.52 (0.38, 0.66)** | **<0.001** |
| Dairy | 253.24 (3.77) | 259.79 (2.35) | 264.16 (3.23) | 266.35 (4.02) | 268.53 (4.89) | **1.09 (0.08, 2.11)** | **<0.05** |
| Eggs | 29.62 (0.42) | 29.69 (0.28) | 29.73 (0.39) | 29.75 (0.48) | 29.77 (0.57) | 0.01 (-0.10, 0.12) | 0.86 |
| Legumes, nuts and seeds | 58.9 (0.84) | 53.77 (0.52) | 50.36 (0.81) | 48.65 (1.02) | 46.94 (1.26) | **-0.85 (-1.11, -0.60)** | **<0.001** |

Values are predicted using survey-weighted generalized linear regression models, with survey cycle treated as a continuous variable and adjusting for total energy intake.

Values represent predicted survey-weighted mean grams consumed per capita (±SE), holding total energy intake at the average 1,814 kcal/day (±8.4) across all survey cycles.

*Total meat corresponds to the sum of unprocessed red meat and processed meat consumed by each participant.

**Survey-weighted generalized linear regression models included survey cycle as a continuous variable and adjusted for total energy intake.

†Bolded p-values are significant at the α = 0.05 level.

# **Supplemental Table 3 – Trends in seafood consumption (grams per survey-cycle) in Mexican population adjusted for total energy intake stratified by age group, sex, educational attainment of head of household, socioeconomic status, indigenous status, area of residence, ENSANUT 2006 - 2020**

|  | **2006**  **n = 38,775** | **2012**  **n = 6,729** | **2016**  **n = 14,537** | **2018**  **n = 23,516** | **2020**  **n = 2,033** | **Average change per survey cycle**  **(95% CI) *** | **P-value†** |
| --- | --- | --- | --- | --- | --- | --- | --- |
| **Age group** |  |  |  |  |  |  |  |
| 0 -5 years | 3.3 (0.2) | 3.4 (0.2) | 3.4 (0.2) | 3.5 (0.2) | 3.5 (0.3) | 0.02 ( -0.04, 0.07) |  |
| 6-11 years | 3.5 (0.2) | 4.2 (0.1) | 4.6 (0.2) | 4.9 (0.2) | 5.1 (0.3) | **0.12 (0.06, 0.17)** |  |
| 12-19 years | 7.2 (0.4) | 7.2 (0.3) | 7.3 (0.4) | 7.3 (0.5) | 7.3 (0.6) | 0.01 ( -0.09, 0.11) | **<0.001** |
| 20-59 years | 10.1 (0.4) | 8.7 (0.2) | 7.7 (0.3) | 7.2 (0.4) | 6.7 (0.5) | **-0.24 ( -0.34, -0.14)** |  |
| 60 + years | 9.5 (0.6) | 8.2 (0.4) | 7.3 (0.4) | 6.9 (0.5) | 6.4 (0.6) | **-0.22 ( -0.36, -0.07)** |  |
| **Sex** |  |  |  |  |  |  |  |
| Female | 7.4 (0.2) | 6.7 (0.1) | 6.3 (0.2) | 6.1 (0.2) | 5.9 (0.3) | -0.11 ( -0.16, -0.05) | 0.44 |
| Male | 8.6 (0.3) | 7.8 (0.2) | 7.2 (0.3) | 6.9 (0.4) | 6.6 (0.5) | -0.15 ( -0.24, -0.05) |  |
| **Educational Attainment of HoH** |  |  |  |  |  |  |  |
| None to middle school | 7.1 (0.2) | 6.2 (0.1) | 5.6 (0.2) | 5.3 (0.2) | 5 (0.3) | -0.15 ( -0.2, -0.09) |  |
| High School, trade or professional training | 9.6 (0.6) | 8.6 (0.3) | 8 (0.3) | 7.6 (0.4) | 7.3 (0.5) | -0.16 ( -0.29, -0.04) | 0.97 |
| Bachelor’s or higher | 14 (1.2) | 13.1 (0.8) | 12.5 (0.8) | 12.2 (0.9) | 11.9 (1.1) | -0.15 ( -0.39, 0.09) |  |
| **Socioeconomic Status** |  |  |  |  |  |  |  |
| Low | 7.3 (0.4) | 5.9 (0.2) | 5 (0.3) | 4.6 (0.3) | 4.1 (0.4) | **-0.23 ( -0.32, -0.14)** |  |
| Middle | 6.8 (0.3) | 6.4 (0.2) | 6.1 (0.3) | 5.9 (0.3) | 5.8 (0.4) | -0.07 ( -0.16, 0.01) | **<0.05** |
| High | 9.3 (0.4) | 8.6 (0.2) | 8.1 (0.3) | 7.8 (0.3) | 7.6 (0.4) | **-0.12 ( -0.2, -0.04)** |  |
| **Indigenous** |  |  |  |  |  |  |  |
| No | 9.3 (0.4) | 8.6 (0.2) | 8.1 (0.3) | 7.8 (0.3) | 7.6 (0.4) | -0.12 ( -0.18, -0.07) |  |
| Yes | 5.1 (0.5) | 4.1 (0.3) | 3.4 (0.3) | 3.1 (0.4) | 2.7 (0.4) | -0.17 ( -0.28, -0.06) | 0.42 |
| **Area of residence** |  |  |  |  |  |  |  |
| Rural | 6.3 (0.4) | 5.7 (0.2) | 5.3 (0.3) | 5.1 (0.4) | 4.9 (0.5) | -0.09 ( -0.2, 0.01) | 0.51 |
| Urban | 8.5 (0.3) | 7.6 (0.2) | 7.1 (0.2) | 6.8 (0.2) | 6.6 (0.3) | -0.13 ( -0.2, -0.07) |  |

Values are predicted using survey-weighted generalized linear regression models, with survey cycle treated as a continuous variable, and adjusting for sociodemographic group and total energy intake. Values represent survey-weighted mean grams consumed per capita (±SE) for each sociodemographic group, holding total energy intake within the sociodemographic group constant across all survey cycles. HoH refers to head of household.

*Average change per survey cycle per sociodemographic group is calculated as the linear combination of interaction terms derived from survey-weighted generalized linear regressions adjusted for survey year, sociodemographic factor, and total energy intake in addition to the interaction term. Interaction terms were sociodemographic factors multiplied by survey year to test for varying trends by sociodemographic factor.

†P-values were derived from a Wald test of interaction terms. Bolded p-values are significant at the α = 0.05 level.

# **Supplemental Table 4 – Trends in poultry consumption (g per survey-cycle) in Mexican population adjusted by total energy intake stratified by age group, sex, educational attainment of head of household, socioeconomic status, indigenous status, area of residence, ENSANUT 2006 - 2020**

|  | **2006**  **n = 38,775** | **2012**  **n = 6,729** | **2016**  **n = 14,537** | **2018**  **n = 23,516** | **2020**  **n = 2,033** | **Average change per survey cycle**  **(95% CI) *** | **P-value†** |
| --- | --- | --- | --- | --- | --- | --- | --- |
| **Age group** |  |  |  |  |  |  |  |
| 0 -5 years | 14.5 (0.7) | 18.2 (0.4) | 20.7 (0.7) | 21.9 (0.9) | 23.1 (1.1) | **0.61 (0.39, 0.84)** |  |
| 6-11 years | 15.7 (0.5) | 19.4 (0.3) | 21.9 (0.5) | 23.2 (0.6) | 24.4 (0.7) | **0.62 (0.49, 0.76)** |  |
| 12-19 years | 16.1 (0.7) | 21.7 (0.4) | 25.5 (0.6) | 27.3 (0.8) | 29.2 (1) | **0.93 (0.72, 1.14)** | **<0.01** |
| 20-59 years | 22 (0.8) | 24 (0.5) | 25.3 (1) | 26 (1.3) | 26.7 (1.7) | **0.33 (0.02, 0.65)** |  |
| 60 + years | 21.9 (1.4) | 23.2 (0.7) | 24.1 (1.3) | 24.5 (1.7) | 25 (2.1) | 0.22 ( -0.22, 0.66) |  |
| **Sex** |  |  |  |  |  |  |  |
| Female | 18.5 (0.5) | 21.6 (0.3) | 23.7 (0.6) | 24.8 (0.7) | 25.8 (0.9) | 0.52 (0.35, 0.69) |  |
| Male | 19.9 (0.7) | 23 (0.4) | 25 (0.8) | 26 (1) | 27 (1.2) | 0.51 (0.27, 0.75) | 0.95 |
| **Educational Attainment of HoH** |  |  |  |  |  |  |  |
| None to middle school | 18 (0.4) | 20.5 (0.3) | 22.2 (0.5) | 23 (0.7) | 23.9 (0.8) | 0.42 (0.26, 0.58) |  |
| High School, trade or professional training | 22.4 (1.4) | 26.6 (0.7) | 29.3 (1.1) | 30.7 (1.4) | 32.1 (1.8) | 0.69 (0.29, 1.08) | 0.43 |
| Bachelor’s or higher | 25.3 (1.4) | 28.5 (0.8) | 30.7 (1) | 31.8 (1.2) | 32.9 (1.4) | 0.55 (0.22, 0.87) |  |
| **Socioeconomic Status** |  |  |  |  |  |  |  |
| Low | 16.4 (0.8) | 18.7 (0.5) | 20.3 (0.9) | 21 (1.2) | 21.8 (1.4) | 0.38 (0.1, 0.67) |  |
| Middle | 17.5 (0.9) | 20.7 (0.5) | 22.9 (0.9) | 24 (1.2) | 25.1 (1.5) | 0.54 (0.22, 0.86) | 0.71 |
| High | 22.5 (0.6) | 25.6 (0.4) | 27.7 (0.6) | 28.7 (0.8) | 29.7 (0.9) | 0.51 (0.33, 0.7) |  |
| **Indigenous** |  |  |  |  |  |  |  |
| No | 19.1 (0.4) | 22.4 (0.3) | 24.6 (0.5) | 25.7 (0.6) | 26.8 (0.7) | **0.55 (0.4, 0.69)** | **<0.001** |
| Yes | 18.7 (1.1) | 18.7 (0.8) | 18.7 (1) | 18.7 (1.1) | 18.7 (1.3) | 0.0 ( -0.24, 0.24) |  |
| **Area of residence** |  |  |  |  |  |  |  |
| Rural | 14.6 (0.5) | 17.1 (0.3) | 18.7 (0.4) | 19.6 (0.5) | 20.4 (0.7) | 0.41 (0.27, 0.56) | 0.28 |
| Urban | 20.6 (0.5) | 23.8 (0.3) | 26 (0.6) | 27 (0.7) | 28.1 (0.9) | 0.54 (0.36, 0.71) |  |

Values are predicted using survey-weighted generalized linear regression models, with survey cycle treated as a continuous variable, and adjusting for sociodemographic group and total energy intake. Values represent survey-weighted mean grams consumed per capita (±SE) for each sociodemographic group, holding total energy intake within the sociodemographic group constant across all survey cycles. HoH refers to head of household.

** Average change per survey cycle per sociodemographic group is calculated as the linear combination of interaction terms derived from survey-weighted generalized linear regressions adjusted for survey year, sociodemographic factor, and total energy intake in addition to the interaction term. Interaction terms were sociodemographic factors multiplied by survey year to test for varying trends by sociodemographic factor.

†P-values were derived from a Wald test of interaction terms. Bolded p-values are significant at the α = 0.05 level.

# **Supplemental Table 5 – Trends in dairy consumption (g per survey-cycle) in Mexican population adjusted for total energy intake stratified by age group, sex, educational attainment of head of household, socioeconomic status, indigenous status, area of residence, ENSANUT 2006 - 2020**

|  | **2006**  **n = 38,775** | **2012**  **n = 6,729** | **2016**  **n = 14,537** | **2018**  **n = 23,516** | **2020**  **n = 2,033** | **Average change per survey cycle**  **(95% CI) *** | **P-value†** |
| --- | --- | --- | --- | --- | --- | --- | --- |
| Age group |  |  |  |  |  |  |  |
| 0 -5 years | 374.6 (9.9) | 438.3 (6.1) | 480.8 (7.9) | 502.1 (9.6) | 523.3 (11.7) | **10.63 (8.15, 13.1)** |  |
| 6-11 years | 346.5 (7) | 351.3 (4.4) | 354.6 (4.9) | 356.2 (5.9) | 357.8 (7) | 0.81 ( -0.74, 2.36) |  |
| 12-19 years | 250.9 (6.2) | 253.4 (3.7) | 255.1 (4.9) | 255.9 (6.1) | 256.8 (7.4) | 0.42 ( -1.18, 2.01) | **<0.001** |
| 20-59 years | 230.4 (4.7) | 197.8 (3) | 176.1 (5.1) | 165.2 (6.4) | 154.3 (7.9) | **-5.43 ( -7, -3.86)** |  |
| 60 + years | 277.5 (12.3) | 246.2 (7.4) | 225.4 (9.3) | 215 (11.4) | 204.6 (13.9) | **-5.2 ( -8.22, -2.18)** |  |
| **Sex** |  |  |  |  |  |  |  |
| Female | 255.9 (4.2) | 248.5 (2.5) | 243.6 (3.2) | 241.1 (4) | 238.6 (4.9) | **-1.24 ( -2.3, -0.18)** | **<0.001** |
| Male | 248.7 (5.9) | 274.2 (3.7) | 291.3 (5.9) | 299.8 (7.4) | 308.3 (9.1) | **4.25 (2.43, 6.08)** |  |
| **Educational Attainment of HoH** |  |  |  |  |  |  |  |
| None to middle school | 231.8 (4) | 237.9 (2.5) | 241.9 (3.7) | 243.9 (4.6) | 245.9 (5.6) | 1 (-0.13 , 2.13) |  |
| High School, trade or professional training | 324.9 (9.5) | 316.6 (5.2) | 311 (7.1) | 308.2 (9.1) | 305.4 (11.3) | -1.39 (-3.91 , 1.13) | 0.2 |
| Bachelor’s or higher | 350.8 (13.5) | 349.6 (8.1) | 348.7 (10.5) | 348.3 (13.1) | 347.9 (16) | -0.21 (-3.66 , 3.24) |  |
| **Socioeconomic Status** |  |  |  |  |  |  |  |
| Low | 218.7 (7.1) | 223.4 (4.3) | 226.6 (6.9) | 228.2 (8.8) | 229.7 (10.8) | 0.79 ( -1.41, 2.98) |  |
| Middle | 234.8 (5.7) | 250.5 (3.5) | 260.9 (5.1) | 266.2 (6.5) | 271.4 (7.9) | **2.61 (0.98, 4.25)** | **<0.05** |
| High | 292.6 (5.5) | 290.6 (3.5) | 289.3 (4.9) | 288.6 (6) | 287.9 (7.3) | -0.33 ( -1.81, 1.15) |  |
| **Indigenous** |  |  |  |  |  |  |  |
| No | 261.8 (3.9) | 267.1 (2.4) | 270.6 (3.3) | 272.3 (4.2) | 274.1 (5.1) | 0.88 ( -0.17, 1.93) |  |
| Yes | 106.2 (7.3) | 123 (5.4) | 134.3 (6.8) | 139.9 (8.1) | 145.6 (9.6) | 2.81 (1.01, 4.62) | 0.07 |
| **Region of Residence** |  |  |  |  |  |  |  |
| Rural | 164.1 (4.8) | 195 (3) | 215.6 (3.9) | 225.9 (4.8) | 236.1 (5.9) | **5.15 (3.91, 6.39)** | **<0.001** |
| Urban | 280.9 (4.4) | 279.9 (2.8) | 279.2 (4) | 278.8 (5) | 278.5 (6.1) | -0.18 ( -1.41, 1.06) |  |

Values are predicted using survey-weighted generalized linear regression models, with survey cycle treated as a continuous variable, and adjusting for sociodemographic group and total energy intake. Values represent survey-weighted mean grams consumed per capita (±SE) for each sociodemographic group, holding total energy intake within the sociodemographic group constant across all survey cycles. HoH refers to head of household.

** Average change per survey cycle per sociodemographic group is calculated as the linear combination of interaction terms derived from survey-weighted generalized linear regressions adjusted for survey year, sociodemographic factor, and total energy intake in addition to the interaction term. Interaction terms were sociodemographic factors multiplied by survey year to test for varying trends by sociodemographic factor.

†P-values were derived from a Wald test of interaction terms. Bolded p-values are significant at the α = 0.05 level.

# **Supplemental Table 6 – Trends in egg consumption (g per survey-cycle) in Mexican population adjusted for total energy intake stratified by age group, sex, educational attainment of head of household, socioeconomic status, indigenous status, area of residence, ENSANUT 2006 - 2020**

|  | **2006**  **n = 38,775** | **2012**  **n = 6,729** | **2016**  **n = 14,537** | **2018**  **n = 23,516** | **2020**  **n = 2,033** | **Average change per survey cycle**  **(95% CI) *** | **P-value†** |
| --- | --- | --- | --- | --- | --- | --- | --- |
| **Age group** |  |  |  |  |  |  |  |
| 0 -5 years | 24 (0.8) | 25.6 (0.5) | 26.6 (0.6) | 27.2 (0.8) | 27.7 (1) | **0.26 (0.05, 0.47)** |  |
| 6-11 years | 26 (0.6) | 28.3 (0.4) | 29.8 (0.5) | 30.6 (0.6) | 31.4 (0.7) | **0.39 (0.24, 0.53)** |  |
| 12-19 years | 29.7 (0.8) | 31.7 (0.5) | 33 (0.7) | 33.6 (0.8) | 34.2 (1) | **0.32 (0.12, 0.53)** | **<0.001** |
| 20-59 years | 33.7 (0.7) | 31.2 (0.5) | 29.5 (0.8) | 28.6 (1) | 27.8 (1.2) | **-0.43 ( -0.66, -0.2)** |  |
| 60 + years | 25.6 (1.2) | 25.6 (0.7) | 25.6 (0.9) | 25.6 (1.2) | 25.6 (1.5) | 0.0 ( -0.31, 0.32) |  |
| **Sex** |  |  |  |  |  |  |  |
| Female | 26.6 (0.5) | 26.2 (0.3) | 26 (0.4) | 25.9 (0.5) | 25.8 (0.6) | -0.06 ( -0.18, 0.07) |  |
| Male | 33.1 (0.7) | 34 (0.4) | 34.6 (0.7) | 34.8 (0.8) | 35.1 (1) | 0.14 ( -0.06, 0.34) | 0.09 |
| **Educational Attainment of HoH** |  |  |  |  |  |  |  |
| None to middle school | 30.3 (0.5) | 29.3 (0.3) | 28.6 (0.4) | 28.2 (0.5) | 27.9 (0.7) | **-0.17 ( -0.3, -0.04)** |  |
| High School, trade professional training | 27 (1.1) | 30.3 (0.6) | 32.5 (0.8) | 33.6 (1.1) | 34.7 (1.3) | **0.55 (0.26, 0.84)** | **<0.001** |
| Bachelor’s or higher | 27.4 (1.4) | 30.5 (0.8) | 32.6 (1.2) | 33.6 (1.6) | 34.6 (2) | **0.52 ( 0.1, 0.94)** |  |
| **Socioeconomic Status** |  |  |  |  |  |  |  |
| Low | 31.6 (0.8) | 29.3 (0.5) | 27.7 (0.8) | 26.9 (1) | 26.2 (1.3) | **-0.39 ( -0.64, -0.14)** |  |
| Middle | 29.4 (0.7) | 29.7 (0.5) | 29.9 (0.6) | 30 (0.8) | 30.1 (1) | 0.05 ( -0.15, 0.24) | **<0.01** |
| High | 28.7 (0.6) | 29.8 (0.4) | 30.6 (0.6) | 31 (0.7) | 31.4 (0.9) | **0.19 (0.02, 0.36)** |  |
| **Indigenous** |  |  |  |  |  |  |  |
| No | 29.5 (0.4) | 29.7 (0.3) | 29.9 (0.4) | 29.9 (0.5) | 30 (0.6) | 0.03 ( -0.09, 0.15) |  |
| Yes | 30.6 (1.2) | 28.4 (0.9) | 26.9 (1.1) | 26.1 (1.3) | 25.4 (1.5) | **-0.37 ( -0.67, -0.08)** | **<0.05** |
| **Region of residence** |  |  |  |  |  |  |  |
| Rural | 29 (0.6) | 28.2 (0.4) | 27.7 (0.5) | 27.4 (0.6) | 27.2 (0.7) | -0.13 ( -0.28, 0.02) | 0.1 |
| Urban | 29.9 (0.5) | 30.1 (0.3) | 30.3 (0.5) | 30.4 (0.6) | 30.5 (0.7) | 0.05 ( -0.09, 0.19) | 0.1 |

Values are predicted using survey-weighted generalized linear regression models, with survey cycle treated as a continuous variable, and adjusting for sociodemographic group and total energy intake. Values represent survey-weighted mean grams consumed per capita (±SE) for each sociodemographic group, holding total energy intake within the sociodemographic group constant across all survey cycles. HoH refers to head of household.

* Average change per survey cycle per sociodemographic group is calculated as the linear combination of interaction terms derived from survey-weighted generalized linear regressions adjusted for survey year, sociodemographic factor, and total energy intake in addition to the interaction term. Interaction terms were sociodemographic factors multiplied by survey year to test for varying trends by sociodemographic factor.

†P-values were derived from a Wald test of interaction terms. Bolded p-values are significant at the α = 0.05 level.

# **Supplemental Table 7 – Trends in legumes, nuts and seeds consumption (g per survey-cycle) in Mexican population adjusted for total energy intake stratified by age group, sex, educational attainment of head of household, socioeconomic status, indigenous status, area of residence, ENSANUT 2006 - 2020**

|  | **2006**  **n = 38,775** | **2012**  **n = 6,729** | **2016**  **n = 14,537** | **2018**  **n = 23,516** | **2020**  **n = 2,033** | **Average change per survey cycle**  **(95% CI) *** | **P-value†** |
| --- | --- | --- | --- | --- | --- | --- | --- |
| **Age group** |  |  |  |  |  |  |  |
| 0 -5 years | 34.5 (1) | 34.6 (0.6) | 34.7 (0.8) | 34.7 (0.9) | 34.8 (1.2) | 0.02 ( -0.24, 0.28) |  |
| 6-11 years | 39.3 (1.1) | 41.8 (0.7) | 43.5 (0.9) | 44.3 (1.2) | 45.2 (1.4) | **0.42 (0.13, 0.72)** |  |
| 12-19 years | 57.7 (1.3) | 53.8 (0.8) | 51.1 (1) | 49.8 (1.2) | 48.5 (1.5) | **-0.66 ( -0.98, -0.34)** | **<0.001** |
| 20-59 years | 68.6 (1.5) | 59.8 (1) | 53.9 (2.1) | 51 (2.7) | 48 (3.3) | **-1.47 ( -2.1, -0.83)** |  |
| 60 + years | 67.3 (1.9) | 60.2 (1.1) | 55.5 (1.5) | 53.1 (1.9) | 50.7 (2.4) | **-1.18 ( -1.7, -0.67)** |  |
| **Sex** |  |  |  |  |  |  |  |
| Female | 57.3 (0.9) | 51.4 (0.6) | 47.4 (0.7) | 45.5 (0.9) | 43.5 (1.1) | -0.99 ( -1.2, -0.77) |  |
| Male | 60.9 (1.4) | 56.8 (0.8) | 54.1 (1.6) | 52.8 (2.1) | 51.4 (2.6) | -0.68 ( -1.19, -0.16) | 0.3 |
| **Educational Attainment of HoH** |  |  |  |  |  |  |  |
| None to middle school | 61.7 (1) | 56.5 (0.6) | 53.1 (1.1) | 51.3 (1.4) | 49.6 (1.7) | -0.86 ( -1.2, -0.52) |  |
| High School, trade or professional training | 48.5 (1.6) | 45.6 (1.1) | 43.7 (1.4) | 42.7 (1.7) | 41.8 (2.1) | -0.48 ( -0.89, -0.07) | 0.2 |
| Bachelor’s or higher | 46.4 (1.9) | 44 (1.2) | 42.4 (1.4) | 41.6 (1.6) | 40.8 (2) | -0.4 ( -0.83, 0.04) |  |
| **Socioeconomic Status** |  |  |  |  |  |  |  |
| Low | 65.3 (2.2) | 60.4 (1.2) | 57.1 (2.6) | 55.5 (3.5) | 53.9 (4.3) | -0.82 ( -1.68, 0.04) |  |
| Middle | 57.6 (1.2) | 53.4 (0.7) | 50.6 (1) | 49.2 (1.3) | 47.8 (1.6) | -0.7 ( -1.02, -0.37) | 0.8 |
| High | 54.6 (1) | 49.6 (0.7) | 46.2 (0.9) | 44.5 (1) | 42.8 (1.2) | -0.84 ( -1.09, -0.6) |  |
| **Indigenous** |  |  |  |  |  |  |  |
| No | 58.1 (0.9) | 53.3 (0.5) | 50 (0.8) | 48.4 (1.1) | 46.8 (1.3) | **-0.81 ( -1.07, -0.54)** | **<0.05** |
| Yes | 71.9 (2.7) | 63.1 (1.7) | 57.1 (2) | 54.2 (2.3) | 51.2 (2.8) | **-1.48 ( -2.09, -0.87)** |  |
| **Region of Residence** |  |  |  |  |  |  |  |
| Rural | 73.8 (1.6) | 65 (1) | 59.2 (1) | 56.3 (1.3) | 53.4 (1.5) | **-1.46 ( -1.81, -1.11)** | **<0.001** |
| Urban | 54.2 (0.9) | 50.3 (0.6) | 47.7 (1) | 46.4 (1.3) | 45 (1.6) | **-0.65 ( -0.96, -0.35)** |  |

Values are predicted using survey-weighted generalized linear regression models, with survey cycle treated as a continuous variable, and adjusting for sociodemographic group and total energy intake. Values represent survey-weighted mean grams consumed per capita (±SE) for each sociodemographic group, holding total energy intake within the sociodemographic group constant across all survey cycles. HoH refers to head of household.

*Average change per survey cycle per sociodemographic group is calculated as the linear combination of interaction terms derived from survey-weighted generalized linear regressions adjusted for survey year, sociodemographic factor, and total energy intake in addition to the interaction term. Interaction terms were sociodemographic factors multiplied by survey year to test for varying trends by sociodemographic factor.

†P-values were derived from a Wald test of interaction terms. Bolded p-values are significant at the α = 0.05 level.

**Supplemental Table 8 – Trends in meat and other protein-rich food group consumption among total meat consumers in Mexican population, all ages, ENSANUT 2006 - 2020**

|  | **2006**  **n = 33,551** | **2012**  **n = 6,078** | **2016**  **n = 12,555** | **2018**  **n = 21,226** | **2020**  **n = 1,881** | **Average change per survey cycle**  **(95% CI)** † | **P-trend**† |
| --- | --- | --- | --- | --- | --- | --- | --- |
| Unprocessed Red Meat | 34.17 (0.63) | 32.45 (0.35) | 31.3 (0.68) | 30.73 (0.89) | 30.15 (1.1) | **-0.29 (-0.51, -0.06)** | **<0.05** |
| Processed Meat | 16.56 (0.28) | 16.73 (0.16) | 16.85 (0.23) | 16.91 (0.29) | 16.97 (0.35) | 0.03 (-0.05, 0.10) | 0.44 |
| Total Meat* | 50.73 (0.73) | 49.18 (0.41) | 48.15 (0.76) | 47.64 (0.99) | 47.12 (1.23) | **-0.26 (-0.51, -0.01)** | **<0.05** |
| Seafood | 8.02 (0.23) | 7.33 (0.14) | 6.87 (0.18) | 6.64 (0.22) | 6.41 (0.26) | **-0.11 (-0.17, -0.06)** | **<0.001** |
| Poultry | 19.18 (0.46) | 22.46 (0.27) | 24.64 (0.46) | 25.74 (0.58) | 26.83 (0.72) | **0.55 (0.40, 0.69)** | **<0.001** |
| Dairy | 259.32 (3.88) | 265.5 (2.3) | 269.61 (3.25) | 271.67 (4.09) | 273.73 (5.02) | 1.03 (-0.03, 2.09) | 0.06 |
| Eggs | 29.3 (0.45) | 29.77 (0.29) | 30.08 (0.4) | 30.23 (0.49) | 30.39 (0.6) | 0.08 (-0.04, 0.20) | 0.21 |
| Legumes, nuts and seeds | 56.9 (0.87) | 52.21 (0.5) | 49.08 (0.82) | 47.52 (1.06) | 45.96 (1.31) | **-0.78 (-1.05, -0.51)** | **<0.001** |

Values are predicted using survey-weighted generalized linear regression models, with survey cycle treated as a continuous variable and adjusted for total energy intake among total meat consumers. Values represent predicted survey-weighted mean grams consumed among total meat consumers (±SE), holding total energy intake at the average 1,814 kcal/day (±8.4) across all survey cycles.

*Total meat corresponds to the sum of unprocessed red meat and processed meat consumed by each participant.

†Survey-weighted generalized linear regression models included survey cycle as a continuous variable and adjusted for total energy intake. Bolded p-values are significant at the α = 0.05 level.

# **Supplementary Table 9 - Survey-weighted red and processed meat percent contribution to total nutrient intake per capita by age group, ENSANUT 2016**

|  | **0-5 years** | **6-11 years** | **12-19 years** | **20-59 years** | **60+ years** | **P-value** |
| --- | --- | --- | --- | --- | --- | --- |
|  | **Unprocessed red meat** | | | | |  |
| Heme Iron | **15.0 (2.3)** | **20.6 (2.0)** | **26.5 (2.4)** | **24.5 (2.1)** | **21.4 (4.6)** | **<0.001** |
| Vitamin B12 | **9.1 (1.3)** | **14.2 (1.3)** | **17.8 (1.4)** | **19.1 (1.6)** | **15.7 (3.1)** | **<0.001** |
|  | **Processed meat** | | | | | |
| Heme Iron | **26.0 (2.8)** | **34.8 (3.1)** | **29.8 (2.2)** | **24.8 (1.9)** | **14.6 (3.3)** | **<0.001** |
| Vitamin B12 | **7.8 (1.2)** | **10.8 (1.6)** | **12.5 (2.0)** | **9.7 (1.3)** | **7.2 (1.9)** | **<0.01** |

Values are survey-weighted percent contributions to total nutrient intake per capita, % (SE).

P-Values are based on Kruskal-Walli’s test and bolded values are significant at the α = 0.05 level.

# **Supplementary Table 9 (cont.) - Survey-weighted red and processed meat percent contribution to total nutrient intake per capita by sex and indigenous status, ENSANUT 2016**

|  | **Sex** | | | **Indigenous** | | |
| --- | --- | --- | --- | --- | --- | --- |
|  | **Male** | **Female** | **P-Value** | **No** | **Yes** | **P-Value** |
|  | **Unprocessed Red meat** | | | **Unprocessed Red meat** | | |
| Heme Iron | 24.0 (2.1) | 22.1 (1.6) | 0.6 | **23.4 (1.4)** | **15.6 (4.3)** | **0.02** |
| Vitamin B12 | 17.3 (1.4) | 16.4 (1.3) | 0.7 | **17.1 (1.0)** | **13.5 (3.7)** | **0.04** |
|  | **Processed Meat** | | | **Processed Meat** | | |
| Heme Iron | 25.1 (1.9) | 26.5 (1.8) | 0.5 | **26.8 (1.4)** | **8.2 (1.9)** | **<0.001** |
| Vitamin B12 | 9.7 (1.2) | 9.9 (1.2) | 0.9 | **10.1 (0.8)** | **3.5 (1.0)** | **<0.001** |

Values are survey-weighted percent contributions to total nutrient intake per capita, % (SE).

P-Values are based on Kruskal-Wallis test and bolded values are significant at the α = 0.05 level.

# **Supplementary Table 9 (cont.) - Survey-weighted red and processed meat percent contribution to total nutrient intake per capita by socioeconomic status, all ages, ENSANUT 2016**

|  | **Low Income** | **Middle Income** | **High Income** | **P-Value** |
| --- | --- | --- | --- | --- |
|  |  | **Red Meat** |  |  |
| Heme Iron | 20.7 (2.5) | 22.3 (1.9) | 24.3 (2.0) | 0.11 |
| Vitamin B12 | 15.2 (1.5) | 16.4 (1.5) | 17.8 (1.4) | 0.05 |
| **Processed Meat** | | | | |
| Heme Iron | **17.4 (1.8)** | **22.7 (1.6)** | **30.9 (2.2)** | **<0.001** |
| Vitamin B12 | **8.3 (1.2)** | **8.2 (0.9)** | **11.2 (1.4)** | **0.01** |

Values are survey-weighted percent contributions to total nutrient intake per capita, % (SE).

P-Values are based on Kruskal-Wallis test and bolded values are significant at the α = 0.05 level.

# **Supplementary Table 9 (cont.) - Survey-weighted red and processed meat percent contribution to total nutrient intake per capita by educational attainment of head of household, all age groups, ENSANUT 2016**

|  | **None to Middle school** | **High school, trade, or professional training** | **Bachelor’s or higher** | **P-Value** |
| --- | --- | --- | --- | --- |
|  | **Unprocessed red meat** | | |  |
| Heme Iron | 22.2 (1.5) | 23.8 (2.6) | 26.8 (4.3) | 0.3 |
| Vitamin B12 | 16.2 (1.0) | 17.0 (1.9) | 20.8 (3.6) | 0.2 |
|  |  | **Processed meat** |  |  |
| Heme Iron | **23.3 (1.3)** | **31.7 (3.2)** | **33.0 (5.8)** | **<0.05** |
| Vitamin B12 | 9.1 (0.7) | 12.5 (2.5) | 10.1 (3.4) | 0.2 |

Values are survey-weighted percent contributions to total nutrient intake per capita consumers, % (SE).

P-Values are based on Kruskal-Wallis test and bolded values are significant at the α = 0.05 level.

# **Supplemental Table 10 – Trends in consumption of meat and other protein-rich foods among the Mexican population, all ages, ENSANUT 2006 – 2018**

|  | **2006**  **n = 38,775** | **2012**  **n = 6,729** | **2016**  **n = 14,537** | **2018**  **n = 23,516** | **Average change per survey cycle**  **(95% CI)** † | **P-trend†** |
| --- | --- | --- | --- | --- | --- | --- |
| Unprocessed  Red Meat | 29.88 (0.44) | 29.81 (0.3) | 29.76 (0.39) | 29.73 (0.48) | -0.01 (-0.13, 0.10) | 0.82 |
| Processed Meat | 14.1 (0.22) | 15.54 (0.15) | 16.5 (0.19) | 16.98 (0.22) | **0.24 (0.19, 0.29)** | **<0.001** |
| Total Meat* | 43.98 (0.53) | 45.35 (0.36) | 46.26 (0.47) | 46.71 (0.56) | **0.23 (0.09, 0.36)** | **<0.001** |
| Seafood | 7.57 (0.21) | 7.13 (0.14) | 6.84 (0.19) | 6.69 (0.24) | **-0.07 (-0.13, -0.02)** | **0.01** |
| Poultry | 19.14 (0.36) | 21.58 (0.25) | 23.2 (0.35) | 24.02 (0.43) | **0.41 (0.31, 0.51)** | **<0.001** |
| Dairy | 252.51 (3.41) | 254.58 (2.26) | 255.97 (2.72) | 256.66 (3.24) | 0.35 (-0.45, 1.14) | 0.39 |
| Eggs | 28.64 (0.39) | 29.42 (0.28) | 29.94 (0.38) | 30.2 (0.45) | **0.13 (0.03, 0.23)** | **0.01** |
| Legumes, nuts and seeds | 57.81 (0.73) | 52.89 (0.47) | 49.6 (0.57) | 47.96 (0.68) | **-0.82 (-0.99, -0.65)** | **<0.001** |

Values are predicted using survey-weighted generalized linear regression models, with survey cycle treated as a continuous variable and adjusting for total energy intake.

Values represent predicted survey-weighted mean grams consumed per capita (±SE), holding total energy intake at the average 1,766 kcal/day (±6.6) across all survey cycles.

*Total meat corresponds to the sum of unprocessed red meat and processed meat consumed by each participant.

†Survey-weighted generalized linear regression models included survey cycle as a continuous variable and adjusted for total energy intake. Bolded p-values are significant at the α = 0.05 level.
